# Eosinophils in Eosinophilic Esophagitis: The Road to Fibrostenosis is Paved With Good Intentions

**DOI:** 10.3389/fimmu.2020.603295

**Published:** 2020-12-01

**Authors:** Alfred D. Doyle, Mia Y. Masuda, Hirohito Kita, Benjamin L. Wright

**Affiliations:** ^1^ Division of Allergy, Asthma, and Clinical Immunology, Department of Medicine, Mayo Clinic Arizona, Scottsdale, AZ, United States; ^2^ Department of Immunology, Mayo Clinic Arizona, Scottsdale, AZ, United States; ^3^ Division of Pulmonology, Phoenix Children’s Hospital, Phoenix, AZ, United States

**Keywords:** eosinophilic esophagitis, eosinophil, food allergy, oral immunotherapy, epithelial barrier, fibrosis, esophagus

## Abstract

Eosinophilic esophagitis (EoE) is an antigen-driven disease associated with epithelial barrier dysfunction and chronic type 2 inflammation. Eosinophils are the defining feature of EoE histopathology but relatively little is known about their role in disease onset and progression. Classically defined as destructive, end-stage effector cells, eosinophils (a resident leukocyte in most of the GI tract) are increasingly understood to play roles in local immunity, tissue homeostasis, remodeling, and repair. Indeed, asymptomatic esophageal eosinophilia is observed in IgE-mediated food allergy. Interestingly, EoE is a potential complication of oral immunotherapy (OIT) for food allergy. However, we recently found that patients with peanut allergy may have asymptomatic esophageal eosinophilia at baseline and that peanut OIT induces transient esophageal eosinophilia in most subjects. This is seemingly at odds with multiple studies which have shown that EoE disease severity correlates with tissue eosinophilia. Herein, we review the potential role of eosinophils in EoE at different stages of disease pathogenesis. Based on current literature we suggest the following: (1) eosinophils are recruited to the esophagus as a homeostatic response to epithelial barrier disruption; (2) eosinophils mediate barrier-protective activities including local antibody production, mucus production and epithelial turnover; and (3) when type 2 inflammation persists, eosinophils promote fibrosis.

## Introduction

Eosinophilic esophagitis (EoE) is an increasingly prevalent disease entity clinically characterized by symptoms of esophageal dysfunction (1). Endoscopically, EoE is defined by the presence of edema, longitudinal furrows, exudates, and rings and esophageal narrowing in more advanced disease (2). Histopathologic diagnosis requires the presence of esophageal eosinophilia with a tissue eosinophil density ≥15 eos/hpf (1). Patients with EoE are often atopic and up to 70% may have IgE-mediated food allergy (3–5). Treatments for EoE include high-dose proton-pump inhibitors, swallowed topical steroids, dietary elimination, and esophageal dilation (6). While there are no FDA-approved therapies for EoE, a number of clinical trials investigating biologic agents are ongoing (7). In terms of its pathogenesis, EoE is driven primarily by food antigens (8, 9) and less commonly environmental allergens (10, 11); however, EoE does not appear to be IgE-mediated (12). Recent literature suggests that EoE is associated with impaired epithelial barrier function of the esophageal mucosa (13–15). Barrier disruption may alter local antigen processing leading to chronic type 2 inflammation and dysregulation of endogenous protease activity (16, 17). These inflammatory responses (including eosinophilia) may subsequently give rise to a perpetual cycle of remodeling and repair.

## Oral Immunotherapy and the Initiation of EoE

OIT for IgE-mediated food allergy represents a unique vantage point from which to understand the pathogenesis of EoE. OIT is based on the principle that graduated antigen exposure desensitizes acute effector cells (e.g. mast cells, basophils) and modulates antigen-specific T- and B-cell responses allowing for ingestion of pre-defined doses of a triggering food protein (18). Importantly, approximately 50% of subjects receiving OIT develop gastrointestinal symptoms and up to 5% develop EoE (19, 20). Generally, EoE resolves with cessation of OIT; however, some subjects develop persistent disease (21, 22). The prevalence of EoE among patients with food allergy is substantially increased compared to the general population and subjects do not routinely undergo upper endoscopy before starting OIT; therefore, it is difficult to exclude the possibility that OIT subjects have pre-existing subclinical EoE (5).

To address this, we performed a study analyzing longitudinal endoscopic biopsies during a 2-year clinical OIT trial in adults with IgE-mediated peanut allergy (23, 24). We observed that some subjects did, indeed, have asymptomatic esophageal eosinophilia (≥ 15 eos/hpf) at baseline; eosinophils are not present in the normal esophagus. Tissue eosinophilia was associated with mild endoscopic abnormalities (rings, edema, linear furrows) as well as other histopathologic alterations (basal zone hyperplasia). Importantly, while a few subjects had tissue eosinophilia at baseline, all subjects had evidence of dilated intercellular spaces in at least one segment of the esophagus suggesting IgE-mediated food allergy is also associated with epithelial barrier disruption of the esophagus. When participants were followed longitudinally, OIT induced or exacerbated esophageal eosinophilia in almost all subjects. Intriguingly, esophageal eosinophilia was transient in most subjects despite the fact that antigen exposure with OIT was continued. For a majority, tissue eosinophilia was mild and asymptomatic, although one patient developed dysphagia and food impaction and was diagnosed with EoE. The only other subject with persistent esophageal eosinophilia failed the final desensitization challenge after two years of peanut OIT.

The esophageal eosinophilia observed in OIT subjects is usually asymptomatic and transient. However, it is unclear to what extent or when this may occur in EoE subjects. We hypothesize that patients diagnosed with EoE have more profound epithelial barrier impairment and/or dysfunctional wound healing and repair responses that perpetuate type 2 inflammatory responses. It remains an open question as to whether controlled, graduated antigen exposure can desensitize EoE subjects to trigger foods. This is the rationale for recent studies of epicutaneous therapy in EoE (25).

In addition to clinical and histologic features, OIT and EoE subjects share similar immunologic characteristics. For example, OIT induces food-specific IgA and IgG4 responses in saliva and peripheral blood (26, 27). These markers are also increased in the saliva, biopsy homogenates and peripheral blood of patients with EoE (28–30). Importantly, food-specific IgA and IgG4 levels are associated with the development of sustained unresponsiveness to food challenge following OIT (27).

**Figure 1:**
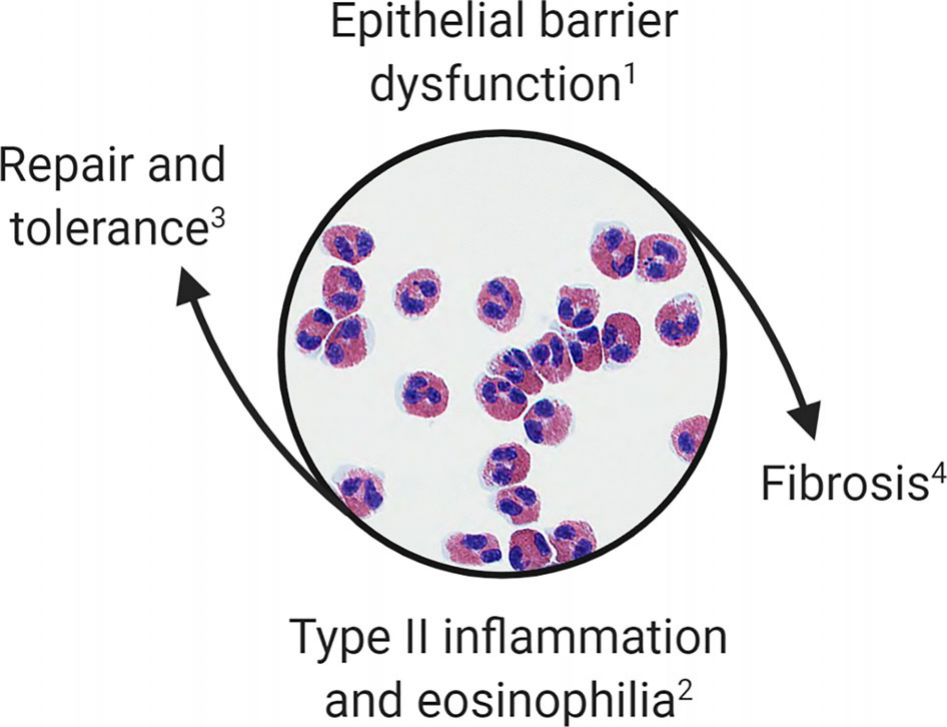
Proposed model of esophageal eosinophilia. Eosinophils are initially recruited to the esophagus to restore barrier function. When inflammatory and remodeling responses become dysregulated eosinophils contribute to type 2 inflammation, worsening of barrier integrity, and fibrosis. This paradigm allows for categorization of (1) patients with IgE-mediated food allergy or subclinical barrier dysfunction; (2) OIT patients, EoEe1, and EoEe2 representing asymptomatic, mild or severe phases of the inflammatory response; (3) OIT patients who successfully develop sustained unresponsiveness or those who naturally outgrow a food allergy; and (4) EoEe3, which is characterized by fibrostenosis.

Overlaps in the clinical and histopathologic features of OIT and EoE subjects suggest that food allergy and EoE exist on the same disease spectrum. Taken together, these observations suggest that: (1) IgE-mediated food allergy, like EoE, is associated with epithelial barrier dysfunction of the esophagus; (2) antigen exposure in this context promotes tissue eosinophilia; (3) esophageal eosinophilia during OIT is often asymptomatic; and (4) antigen-driven tissue eosinophilia can resolve or persist resulting in EoE. We hypothesize that eosinophils are recruited initially during OIT to restore homeostasis; however, when tissue inflammatory and remodeling responses become dysregulated they contribute to EoE pathogenesis (**Figure 1**).

## EoE Endotypes and Disease Progression

Similar to asthma, EoE may be clustered into different endotypes using clinical, endoscopic, histopathologic, and molecular features. A single, multi-site cross-sectional study of endoscopic, histologic, and molecular data from esophageal biopsies identified three discrete EoE endotypes (31). EoE endotype 1 (EoEe1) has a normal endoscopic appearance and is usually steroid-sensitive. Markers of inflammation and epithelial differentiation are relatively unchanged in this endotype. EoE endotype 2 (EoEe2) is usually pediatric-onset, associated with atopy, and steroid-refractory. Gene expression in EoEe2 is notable for marked upregulation of pro-inflammatory mediators (e.g. IL-4 and TSLP). EoE endotype 3 (EoEe3) tends to be adult-onset, non-atopic, and is associated with fibrostenosis and narrow-caliber esophagus. Gene expression in this group denotes loss of epithelial differentiation. Among the endotypes, EoEe1 is the most benign, while EoEe2 is associated with marked type 2 inflammation. Unsupervised clustering of cytokine gene expression suggests these endotypes may be further subdivided into additional subgroups (32). Of note, validation and verification of these endotypes requires further study and natural history studies have not followed EoE cohorts longitudinally in order to understand specific relationships between endotypes; however, these EoE endotypes may represent different points of progression across a continuum of disease. This same principle may be reflected in the fact that children have non-specific gastrointestinal symptoms; whereas, food impaction due to fibrostenosis is much more common in teens and adults.

## Lessons from Targeting Eosinophils and EoE-Like Disease

Unsuccessful clinical trials targeting eosinophils and EoE-like disease provide two lines of evidence supporting the hypothesis that eosinophils may be dispensable in EoE pathology. Given their conspicuous presence in tissue pathology, early clinical trials of biologics in EoE focused on depletion of eosinophils. IL-5 promotes eosinophil maturation, proliferation, activation, and survival (33); therefore, targeting this cytokine pathway was a logical first step. Three anti-IL-5 agents, mepolizumab, reslizumab, and benralizumab, have been developed. Mepolizumab and reslizumab neutralize IL-5 by binding it directly preventing interaction with IL-5Rα. Benralizumab binds to IL-5Rα blocking interaction with IL-5 and promoting antibody-dependent cellular cytotoxicity (ADCC) and deletion of eosinophils. In an alternative strategy, lirentelimab targets siglec-8. Siglec-8 is a transmembrane protein shared by eosinophils and mast cells. Ligation of siglec-8 induces apoptosis of eosinophils and inhibits mast cell activity. Lirentelimab both mediates these activities and induces ADCC of eosinophils (34).

Promising results of an open label trial of mepolizumab in 4 patients with EoE led to the first randomized, double-blind, placebo-controlled (DBPC) trial of mepolizumab in 11 patients (35, 36). The results of this clinical trial were notable for reductions in tissue eosinophil counts but limited clinical improvement in EoE symptoms. A subsequent prospective trial randomized 59 pediatric subjects with EoE to three different dosing arms, using the lowest dose of mepolizumab as a comparator, as opposed to a placebo group (37). While the investigators demonstrated reductions in tissue eosinophilia, again there were no significant differences in clinical symptoms between treatment arms. In the largest randomized DBPC trial of anti-IL-5 therapy in EoE to date, 226 pediatric subjects received reslizumab and, again, significant improvements in clinical symptoms were not seen in the treatment arm compared to placebo (38). Importantly, despite overall reductions in tissue eosinophilia, in each of these trials a majority of subjects did not achieve histologic remission (peak tissue eosinophil counts <15 eos/hpf). Indeed, mepolizumab and reslizumab appear to have greater effects on peripheral blood eosinophils compared to esophageal tissue eosinophils [e.g. 90 vs 55% reduction respectively with mepolizumab (39)]. Together, the outcomes of these trials suggest that eosinophils do not exclusively mediate tissue pathology in EoE or incomplete eosinophil depletion accounts for the persistence of symptoms. Trials of benralizumab and lirentelimab, drugs more likely to completely deplete tissue eosinophils, may be more informative.

Additional evidence that eosinophils may not be the primary driver of clinical symptoms is the existence of EoE-like disease; an entity characterized by EoE symptoms responsive to swallowed topical corticosteroids, but without tissue eosinophilia (40). Kindred of patients with EoE-like disease often reveal multiple family members affected with EoE. Generally, these patients do not have advanced endoscopic findings and their histopathology reveals papillary elongation with increased T cells. These patients are distinguished from EoE patients by reduced eotaxin-3 expression. Finally, patients with EoE-like disease may evolve to develop classical EoE. It is tempting to speculate that EoE-like disease represents yet another endotype on the EoE spectrum. Molecular studies comparing patients with EoE-like disease, food allergy, and EoEe1 are needed to evaluate disease overlap.

## Roles of Eosinophils in EoE

Eosinophils are often considered destructive end-stage effector cells defined by their ability to release toxic granule proteins that can damage surrounding tissue. However, comprehensive reviews of eosinophil activities suggest a much more complex cell with roles in health and disease (41–45). As shown in **Table 1**, eosinophils produce and release various mediators that are involved in inflammation, immunoregulation, and tissue remodeling and repair. In EoE, levels of tissue eosinophilia correlate with disease severity as well as response to treatments (156, 164). On the other hand, eosinophils clearly have a homeostatic or physiologic role as resident cells in the rest of the GI tract and findings from EoE-like disease along with the limited effectiveness of anti-IL-5 therapy suggest a minor role in clinical symptoms in EoE. Moreover, we have recently identified asymptomatic eosinophilia in the esophagus of OIT subjects at baseline. These seemingly divergent observations can be explained by considering the temporal effects of eosinophil activities throughout the progression of EoE. Specifically, we propose that the appearance of eosinophils in the esophagus begins as an extension to their homeostatic function in other GI tissues to enhance barrier function. These eosinophils may become activated to contribute to further protective activities and wound repair and, over time, contribute to disease pathology and fibrosis.

**Table 1:** Protective and pathologic effects of eosinophil-derived mediators.

Eosinophil-derived Mediator	Protective effects	Pathologic effects	References*
IL-1α		Fibrosis	(46–49)
IL-1β	Barrier function: mucosal IgA production wound repair: EMT	Fibrosis	(46, 49–52),
IL-1Ra	Immune tolerance: inhibits IL-1α, IL-1β		(53)
IL-3		Inflammation	(54)
IL-4	Wound repair: EMT	Fibrosis	(46, 49, 55)
IL-5	Eosinophil survival	Eosinophil survival	(49, 56, 57) 32197970
IL-6	Barrier function: mucosal IgA production	Fibrosis	(49, 58, 59)
IL-8		Inflammation angiogenesis	(49, 51, 55, 60–62)
IL-9		Inflammation: mast cell survival and activation Barrier function: decreases adherens and tight junction expression	(63, 64)
IL-10	Immune tolerance: IgG4 production, Treg induction		(65, 66)
IL-13	Wound repair: EMT, synaptopodin barrier function: mucus production, synaptopodin intracellular pH regulation expulsion: epithelial turnover	Inflammation: promotes TARC, MDC, eotaxin barrier dysfunction: synaptopodin, ↓ filaggrin, vimentin, desmoglein, ↑calpain-14 epithelial hyperplasia dilated intercellular space formation fibrosis: activates fibroblasts, stimulates production of TGF-β	(32, 49, 67–73)
IL-17	Antimicrobial	Inflammation	(74)
IL-18		Inflammation	(75–78)
IL-25		Inflammation	(79, 80)
IFNγ	Antimicrobial	Inflammation	(81–83)
TNF-α	Wound repair: EMT	Inflammation angiogenesis	(52, 81, 84, 85)
Osteopontin		Angiogenesis fibrosis	(86)
Amphiregulin	Wound repair immune tolerance: Treg activity	Fibrosis	(87)
APRIL	Plasma cell survival	Plasma cell survival	(59, 88)
BAFF	Plasma cell survival	Plasma cell survival	(88)
SCF		Inflammation: mast cell survival	(89, 90)
TGF-α	Wound repair: EMT	Fibrosis epithelial hyperplasia angiogenesis	(91)
TGF-β	Wound repair: EMT barrier function: mucosal IgA production immune tolerance: Treg induction	Fibrosis: activates fibroblasts, promotes collagen production smooth muscle proliferation/activation epithelial hyperplasia	(46, 92–95)
GM-CSF	Wound repair	Inflammation	(54, 56, 96–98)
VEGF		Angiogenesis Tissue remodeling	(99–101)
FGF-2	Epithelial turnover wound repair	Fibrosis Epithelial hyperplasia smooth muscle activation angiogenesis	(102, 103)
NGF		Nerve growth fibrosis angiogenesis	(104, 105)
HB-EGF	Wound repair	Smooth muscle activation	(106)
PDGF-bb	Wound repair	Angiogenesis smooth muscle activation fibrosis	(107)
Substance P	Wound repair	Pain inflammation angiogenesis	(108)
VIP	Smooth muscle relaxation		(108, 109)
α-defensin	Antimicrobial	Inflammation: innate immune activation	(110, 111)
Angiogenin		Angiogenesis	(99, 112, 113)
MMP-9	Wound repair IL-1β, TGF-β activation	IL-1β, TGF-β activation	(114, 115)
Heparanase	Wound repair	Inflammation angiogenesis	(116–118)
DAO (histaminase)	Resolution of inflammation		(119)
15-lipoxygenase derivatives (e.g ALOX15).	Resolution of inflammation arachidonic acid metabolism		(120–123)
IDO	Immune tolerance	Inflammation angiogenesis	(124, 125)
Superoxide (O_2_^-^)	Antimicrobial	Inflammation	(61)
MBP-1	Antimicrobial epithelial hyperplasia/proliferation (FGF-9)	Cytotoxic barrier dysfunction smooth muscle activation inflammation: mast cell/basophil degranulation fibrosis	(126–132)
EPX	Antimicrobial	Cytotoxic inflammation: mast cell activation fibrosis	(127, 130, 132–134)
ECP	Antimicrobial	Inflammation: mast cell activation neurotoxic cytotoxic	(132, 135)
EDN	Antimicrobial	Inflammation: dendritic cell activation neurotoxic	(132, 136–138)
CLC	Antimicrobial	Inflammation: carrier for other eosinophil granule cationic RNases	(32, 132, 139, 140)
EET’s	Antimicrobial	Contain toxic granules - see above	(79)
PAF		Inflammation	(141)
Thromboxane B2		Smooth muscle activation	(142)
Leukotriene C4	Barrier function: mucus production	Smooth muscle activation inflammation	(143, 144)
PGD2		Inflammation	(145)
PGE2	Resolution of inflammation	Inflammation pain	(142)
PGF2α		Smooth muscle activation inflammation	(146, 147)
Protectin D1	Resolution of inflammation		(148, 149)
CCL17 (TARC)		Inflammation	(150, 151)
CCL22 (MDC)		Inflammation	(150, 151)
CCL5 (RANTES)		Inflammation	(152, 153)
CCL11 (eotaxin-1)		Inflammation	(154)
CXCL5 (ENA-78)		Inflammation angiogenesis	(155)
CXCL1 (GRO-α)	Wound repair	Inflammation angiogenesis	(49, 62, 156–158)
CCL2 (MCP-1)		Inflammation	(159, 160)
CCL23 (MIP-1α)		Inflammation	(49, 84, 161)
CCL4 (MIP-1β)		Inflammation	(62, 159, 162)
CXCL9 (MIG)		Inflammation	(163)
CXCL10 (IP10)		Inflammation	(163)

*Each mediator has references listed that support production by eosinophils. Additional references implicate certain mediators in EoE, though the source may not be identified.

Eosinophil activities in allergic disease are well studied, particularly in asthma, with identified roles for inflammation (e.g. MBP, IL-13), mucus production (IL-13), epithelial damage (MBP, EPX), tissue remodeling/fibrosis (IL-13, TGF-β), and smooth muscle hyperresponsiveness (IL-13, leukotrienes) (165, 166). These pathways have also been observed in EoE by examination of patient biopsies, cell culture experiments, and mouse models. The many potential roles of eosinophils in EoE are well reviewed (67, 167–174). Below we highlight examples of eosinophil activities in barrier maintenance, defense, repair, and fibrosis that suggest esophageal eosinophilia is a protective response that becomes problematic over time.

## Early Phase/Protective Response in EoE

### Recruitment of Eosinophils

The epithelial barrier has been implicated as central to the disease process in EoE (13, 175). While initiating events remain unclear in EoE, environmental insults to the epithelium (e.g. allergens) can trigger the release of inflammatory signaling molecules including TSLP, IL-25, and IL-33 [all shown to be elevated in EoE (79)] that promote a type 2 inflammatory response [e.g. IL-13 production by ILC2s (176, 177)] which leads to production of eosinophil chemotactic factors, particularly eotaxin-3 (178).

### Eosinophils and Epithelial Barrier Maintenance

Eosinophils have been linked with a host of activities that help to protect/restore the epithelial barrier including antimicrobial defenses, remodeling and repair activities, and immune regulation. Mice deficient for eosinophils have shown that under homeostatic conditions eosinophils support mucus-secreting goblet cell numbers in the small intestine (50). Expression of certain mucins has been shown to be upregulated in biopsies of patients with EoE and EoE mouse models [e.g (51, 179).] but further investigation is needed to understand the activities of esophageal glands that are located beyond the reach of these biopsies (i.e. in the lamina propria and submucosa). Interestingly, in our experience with mouse and pig models of EoE, eosinophils tend to accumulate in the lamina propria similar to the rest of the GI tract (unpublished observations). Notably, IL-5 induced esophageal eosinophilia in a transgenic mouse model was not sufficient to induce pathology but with additional stimulus from a hapten increased epithelial layer eosinophilia was observed along with pathologies associated with EoE (180). In humans, a recent retrospective study of esophageal biopsies utilizing specialized forceps that enabled more reliable subepithelial sampling found that one-third of subjects demonstrated greater subepithelial eosinophil density as compared to the epithelium (181, 182). These observations suggest a likely unappreciated level of eosinophils in the esophageal lamina propria. Further investigation is needed to understand the role of subepithelial esophageal eosinophilia in disease pathogenesis.

### Eosinophils and Epithelial Barrier Defense

Eosinophils have been shown to directly mediate host anti-microbial defense activities in the gut. For example, in response to activation with LPS, S. aureus, C5a, or TSLP, eosinophils release eosinophil extracellular traps (EETs). EETs are mitochondrial DNA laced with toxic eosinophil granule proteins (i.e. MBP, EPX, EDN, ECP) that are released into the extracellular space and can bind and kill bacteria. Indeed, hypereosinophilic mice exhibited local extracellular DNA deposition and were protected against sepsis after cecal ligation and puncture (183). Notably, EETs were detected in the esophagus of active EoE subjects (79), suggesting a role for this mechanism in protection against microbes in EoE. In addition, MBP and IL-13 in particular induce epithelial turnover, an effective mechanism for expulsion of organisms/substances and replacement of damaged epithelium (126, 184).

### Eosinophils and Epithelial Barrier Repair/Immune Tolerance

As shown in **Table 1**, eosinophils can produce factors that help to restore the barrier by promoting epithelial to mesenchymal transition (EMT) which facilitates wound repair. These factors may include, but are not limited to, TGF-β (92), MMP-9 (114), IL-4 (185), IL-13 (185), EPX (127), and MBP (92, 93, 186). IL-13 in particular is considered a central mediator in EoE (68, 69) and IL-13 expressing eosinophils have been identified in the esophageal tissue of EoE subjects (185). Notably, IL-33 promotes IL-13 production by eosinophils (70, 187–190). Recently, IL-13 has been shown to upregulate synaptopodin, an actin-associated protein associated with wound healing, and barrier integrity, in the epithelium of EoE subjects (71). Eosinophils can also modulate the immune response to facilitate barrier repair. Indeed, mice deficient for eosinophils have established that eosinophils support IgA production (50, 88), which in turn, is secreted to the lumen to facilitate barrier function. Notably, food-specific IgA is increased in EoE (30). Finally, eosinophils expressing MHCII and CD80 have been identified in EoE subjects (191, 192) and may present antigen to T cells. TGF-β and IL-10 can influence the production of IgA, IgG4, and T regulatory cell responses. Both cytokines are produced by eosinophils in EoE (92, 185) but a mechanistic link remains to be established.

## Chronic Phase/Pathologic Activities in EoE

During the chronic phase of the disease, eosinophil activities may contribute to inflammation, tissue remodeling, and fibrosis. Eosinophil-derived mediators that are helpful in barrier defense and repair can, over time, contribute to these activities.

### Eosinophils and Inflammation

Eosinophils may promote sustained eosinophilic inflammation by production of eosinophil survival factors GM-CSF and IL-5, expression of which has been observed in tissue eosinophils from EoE subjects (185). The eosinophil microenvironment may become problematic for the epithelium with chronic inflammation—for example, eosinophil oxygen metabolism may induce tissue hypoxia resulting in barrier impairment (193). Eosinophils are also a source of IL-9 and have been linked with mast cell numbers in the esophagus (63) which are, in turn, linked with disease severity (194). Interestingly, mast cell numbers are increased in EoE as compared to EoE-like disease (40). In addition, eosinophils produce eicosanoids including PGD2. PGD2 signals through CRTH2, which has been shown to support ILC2 accumulation (195). CRTH2 has also been shown to be expressed by a subset of IL-5 and IL-13 producing Th2 cells in EoE (196). Interestingly, elevated numbers of CRTH2+CD4+ T cells are observed in EoE as compared to EoE-like disease (40). Finally, eosinophil granule proteins including MBP can damage epithelium resulting in increased pro-inflammatory mediators (197) and have been shown to reduce barrier integrity in the colonic epithelium (128). MBP also can induce mast cell and basophil degranulation as well as smooth muscle and fibroblast activation (129, 198–200) thereby contributing to inflammation and fibrosis.

### Eosinophils and Remodeling/Fibrosis

Chronic IL-13-mediated wound healing activities may become problematic. For example, IL-13 induced synaptopodin overexpression has been shown to impair barrier integrity and reduce epithelial differentiation (71). IL-13 is also linked with epithelial barrier disruption by downregulation of epithelial junction molecules and upregulation of the protease calpain-14 (201). Phase 2 trials of biologics targeting IL-13 pathways have demonstrated improvement in endoscopic and histologic findings in EoE (202–204). Data from mouse models of EoE crossed with eosinophil deficient lines suggest a role for eosinophils in hyperplasia and fibrosis in an allergen-driven model (179, 205) while no role was observed in an IL-13 overexpression model (206). Together these findings would be consistent with a role for eosinophil-derived IL-13 in these remodeling activities that are hallmark pathological features of human EoE (206). Notably, eosinophil-derived IL-13 caused extensive remodeling in the mouse lung by promoting MMP-12 production, a mediator identified as elevated in EoE (120, 207). The activities of MMP-12 in human EoE require investigation. Eosinophil-derived factors IL-13 and TGF-β (and others including IL-1β, and IL-4) induce fibroblast to myofibroblast differentiation and eosinophil-derived TGF-β in particular is linked with production of collagen (46, 208, 209). TGF-β also can induce smooth muscle proliferation, hyperplasia, and contraction (210) which may contribute to esophageal dysmotility. Finally, activated eosinophils produce angiogenic factors such as VEGF and nerve remodeling factors such as NGF and EDN which may contribute to nerve growth and cytotoxicity, respectively.

## Discussion

The role(s) of eosinophils remains unclear in EoE. The observations we and others have made show asymptomatic eosinophilia is likely to be a common occurrence. This suggests that, like other areas of the GI tract, eosinophils may promote tissue homeostasis. Eosinophil activities in EoE and other diseases suggest a role for protecting/restoring the barrier. However, if the barrier is not restored it is likely that eosinophils contribute to inflammation and remodeling/fibrosis. Notably, many of the eosinophil-derived mediators discussed herein have wound healing barrier restoring activities in addition to being linked with pathologies associated with chronic inflammation such as fibrosis. Thus, we suggest the road to fibrostenosis is paved with good intentions. These observations also suggest it may be important to target eosinophils based on EoE endotype. Conceivably targeting those with the fibrostenotic EoE (EoEe3) may result in reduced chronic remodeling pathology while sparing subjects in whom eosinophils may primarily benefit esophageal barrier function. In addition, our perspective suggests therapeutic strategies aimed at protecting, improving, or restoring barrier function by promoting homeostatic eosinophil pathways (e.g. mucus and antibody production) may be helpful.

## Author Contributions

AD and BW drafted the manuscript. MM contributed to the content of the table. AD, MM, HK, and BW reviewed and provided critical feedback on the manuscript. All authors contributed to the article and approved the submitted version.

## Funding

This study is supported by the Don and Kathy Levin Family Foundation (ADD, MYM, BLW), Mayo Clinic Foundation (BLW, HK), Phoenix Children’s Hospital Foundation (BLW), Arizona Biomedical Research Consortium (ADHS18-198880) (BLW). Dr. Wright is a scholar in the Consortium of Eosinophilic Gastrointestinal Disease Researchers (CEGIR). CEGIR (U54 AI117804) is part of the Rare Disease Clinical Research Network (RDCRN), an initiative of the Office of Rare Diseases Research (ORDR), NCATS, and is funded through collaboration between NIAID, NIDDK, and NCATS. CEGIR is also supported by patient advocacy groups including American Partnership for Eosinophilic Disorders (APFED), Campaign Urging Research for Eosinophilic Diseases (CURED), and Eosinophilic Family Coalition (EFC). As a member of the RDCRN, CEGIR is also supported by its Data Management and Coordinating Center (DMCC) (U2CTR002818).

## Conflict of Interest

The authors declare that the research was conducted in the absence of any commercial or financial relationships that could be construed as a potential conflict of interest.

## Abbreviations

EoE, Eosinophilic esophagitis; GI, Gastrointestinal; OIT, Oral immunotherapy; FDA, Food and Drug Administration; Ig, Immunoglobulin; EoEe, EoE endotype; IL, Interleukin; TSLP, Thymic stromal lymphopoietin; MBP, Major basic protein; EPX, Eosinophil peroxidase; TGF, Transforming growth factor; ILC, Innate lymphoid cell; LPS, Lipopolysaccharide; S. aureus, Staphylococcus aureus; C5a, Complement component 5a; EETs, Eosinophil extracellular traps; DNA, Deoxyribonucleic acid; EMT, epithelial-mesenchymal transition; MMP, Matrix metalloproteinase; MHC, Major histocompatibility complex; CD, Cluster of differentiation; GM-CSF, Granulocyte-macrophage colony-stimulating factor; PG, Prostaglandin; CRTH2, Prostaglandin D2 receptor; Th, T helper; VEGF, Vascular endothelial growth factor; NGF, Nerve growth factor; EDN, Eosinophil-derived neurotoxin; IFN, Interferon; TNF, Tumor necrosis factor; APRIL, A proliferation-inducing ligand; BAFF, B-cell activating factor; SCF, Stem cell factor; FGF, Fibroblast growth factor; HB-EGF, Heparin-binding EGF-like growth factor; PDGF, Platelet-derived growth factor; VIP, Vasoactive intestinal peptide; DAO, Diamine oxidase; ALOX, arachidonate lipoxygenase; IDO, Indoleamine 2,3-dioxygenase; ECP, Eosinophil cationic protein; PAF, Platelet activating factor; CCL, C-C motif chemokine; CXCL, C-X-C motif chemokine.
